# A Systematic Review of Non-Contact Sensing for Developing a Platform to Contain COVID-19

**DOI:** 10.3390/mi11100912

**Published:** 2020-09-30

**Authors:** Muhammad Bilal Khan, Zhiya Zhang, Lin Li, Wei Zhao, Mohammed Ali Mohammed Al Hababi, Xiaodong Yang, Qammer H. Abbasi

**Affiliations:** 1 School of Electronic Engineering, Xidian University, Xi’an 710071, China; bilal@stu.xidian.edu.cn (M.B.K.); zyzhang@xidian.edu.cn (Z.Z.); mohammed_al-hababi@stu.xidian.edu.cn (M.A.M.A.H.); 2 Department of Electrical and Computer Engineering, COMSATS University Islamabad, Attock Campus, Islamabad 43600, Pakistan; 3 Key Laboratory of Quantum Optics, Shanghai Institute of Optics and Fine Mechanics, Chinese Academy of Sciences, Shanghai 201800, China; 4 School of Mechano-Electronic Engineering, Xidian University, Xi’an 710071, China; weizhao@xidian.edu.cn; 5 School of Engineering, University of Glasgow, Glasgow G12 8QQ, UK; Qammer.abbasi@glasgow.ac.uk

**Keywords:** COVID-19, CSI, non-contact, SDR, Wi-Fi

## Abstract

The rapid spread of the novel coronavirus disease, COVID-19, and its resulting situation has garnered much effort to contain the virus through scientific research. The tragedy has not yet fully run its course, but it is already clear that the crisis is thoroughly global, and science is at the forefront in the fight against the virus. This includes medical professionals trying to cure the sick at risk to their own health; public health management tracking the virus and guardedly calling on such measures as social distancing to curb its spread; and researchers now engaged in the development of diagnostics, monitoring methods, treatments and vaccines. Recent advances in non-contact sensing to improve health care is the motivation of this study in order to contribute to the containment of the COVID-19 outbreak. The objective of this study is to articulate an innovative solution for early diagnosis of COVID-19 symptoms such as abnormal breathing rate, coughing and other vital health problems. To obtain an effective and feasible solution from existing platforms, this study identifies the existing methods used for human activity and health monitoring in a non-contact manner. This systematic review presents the data collection technology, data preprocessing, data preparation, features extraction, classification algorithms and performance achieved by the various non-contact sensing platforms. This study proposes a non-contact sensing platform for the early diagnosis of COVID-19 symptoms and monitoring of the human activities and health during the isolation or quarantine period. Finally, we highlight challenges in developing non-contact sensing platforms to effectively control the COVID-19 situation.

## 1. Introduction

The pandemic of COVID-19 is exponentially spreading all over the world. Due to this exponential increase, many people have been affected or have died, and as a result the entire world is quarantined from each other. As the outbreak continues to evolve, every country’s government is considering options to prevent the spread of the virus to new places by stopping human movement in places where the disease that causes COVID-19 is already circulating [1]. Self-quarantine is the only option to make one’s own and others’ lives safe. The quarantine of a person is the limit of activities or the separation of persons who are not actually ill but who may be exposed to disease or an infectious agent, whereas isolation is the separation of infected or ill persons from others to prevent the spread of infection or contamination. Country authorities should properly communicate to people before implementation of quarantine and take such measures to improve compliance and reduce panic. They must provide people with clear, consistent and up-to-date guidelines and with reliable information about quarantine measures [2]. It is essential to have constructive engagement with communities in case quarantine measures are to be taken. Persons who are quarantined need to be provided with financial and social–psychosocial support, health care and basic needs, including water, food and other life necessities. The requirements of the vulnerable public should be prioritized. Geographic, cultural and economic elements affect the efficiency of quarantine. Prompt judgment of the local context should assess both the potential barriers and drivers of success to quarantine, and they should be used to notify plans for the best applicable and culturally-known measures. In the situation of the present COVID-19 outbreak, the worldwide containment plan consists of rapid laboratory tests to identify confirmed cases and control them, either in a hospital or at home during the isolation period. Any individual in quarantine who feels a febrile illness or respiratory symptoms at any point should be treated and registered as a suspected case of COVID-19 [1]. However, quarantine is the only existing solution to contain the spread of the virus and to maintain the necessities of life. In this regard, scientists, doctors, engineers and many different communities are doing their best to find promising solutions.

In this study, the focus is on the development of a platform that can diagnose early symptoms of COVID-19 and monitor human activities and health during quarantine and isolation periods. Although wearable sensors and camera-based technology are mature solutions for monitoring of human activities and health [3], wearable sensors have direct contact with the human body, so they may become a source of spreading virus; on the other hand, camera-based technology has no direct contact with the human body but still has issues with individual privacy and the monitoring of blind spots [4,5]. Due to these technical and social issues, non-contact sensing platform development is proposed to avoid any kind of contact in monitoring during the COVID-19 pandemic. Recently, extensive research on activity recognition and classification (ARC) of human activities in the field of health care has resulted in promising new solutions [6]. Specifically, device-free ARC-based platforms are becoming popular. A software-defined radio (SDR) technology-based platform was designed for the detection of human activity. This platform can be used for multiple health applications due to flexibility and scalability of the software-defined hardware [7]. The device-free sensing approach is becoming very popular because patients are not required to carry any device, but instead the wireless channel is tagged with devices to capture the required information [8]. Wi-Fi-based devices have grown very rapidly because they are easy to deploy and are cost effective. Recently, wireless channel state information (WCSI) captured by Wi-Fi devices has been widely used for different sensing purposes [9,10,11,12].

Advances in wireless sensing technologies have the potential to reduce the health care services load from hospital to home, thereby securing hospital facilities. The deployment of wireless sensing health monitoring technology is a promising and feasible solution to handle the COVID-19 pandemic. In this review, a comprehensive study of various platforms used for early diagnosis of vital signs, human activity monitoring and health is presented. This study proposes a non-contact platform for the early diagnosis of COVID-19 symptoms and the monitoring of human activities and health during the quarantine or isolation period. The main goal is to identify non-contact sensing technologies used for monitoring human daily activities like sitting, standing, walking, sleeping and eating, and health monitoring, such as respiration rate, heartbeat, fall, sleep disorder and a balanced diet. This study proposes a solution using the existing literature to develop a platform to diagnose early symptoms of COVID-19 and monitor human activities and health during the quarantine and isolation period in a non-contact manner. Although COVID-19 affects different people in different ways, most infected people will develop mild to moderate symptoms including cough, fever and shortness of breath [2]. The review presents the methods of data collection using non-contact sensing technology, the human activity and health condition classification approach and performance achieved by the existing platforms. We categorize the studies into monitoring of human activities and health conditions. Furthermore, the review presents advantages and limitations. Finally, we summarize and explain some challenges to open research problems that require further investigation and improvements.

The following are the contributions of the comprehensive study:

This study provides a road map in developing a COVID-19 pandemic platform for containing the virus.

(1) Systematically review the non-contact sensing platforms used for human activity and health monitoring.
(2) Propose a non-contact sensing platform for the early diagnosis of COVID-19 symptoms and the monitoring of human activities and health during the isolation or quarantine period.
(3) Highlight the challenges, testing environment, performance and optimal solutions to work on deployment.

The rest of paper is organized as follows: Section 2 includes a literature review of the COVID-19 pandemic, the existing non-contact wireless sensing platforms and technology exploited, the monitoring of human activities and health, and the classification approach and accuracy achieved. In Section 3, the proposed platform is described for the early diagnosis of COVID-19 symptoms and monitoring of human activities and health during the isolation or quarantine period. In Section 4, the experimental setup based on both commercial and specialized hardware is presented. In Section 5, the advantages of developing a non-contact WCSI sensing platform for containing COVID-19 are explained. In Section 6, the challenges faced in developing a non-contact sensing platform are discussed. In Section 7, future recommendations and possible solutions are discussed. In Section 8, conclusions on the non-contact sensing platform development for containing the COVID-19 are made. This study used a list of abbreviations, as defined in Table 1.

## 2. Literature Review

In this section, we present a summary on the origination, spreading mechanisms, symptoms and prevention methods of COVID-19. Then follows a systematic review of non-contact sensing platforms for human activities and health monitoring. This review identifies reliable and intelligent existing related work to propose a new platform for the early diagnosis of COVID-19 symptoms and monitoring of human activities and health to protect human life.

### 2.1. Covid-19 Summary

COVID-19 is a respiratory disease caused by severe acute respiratory syndrome coronavirus-2 (SARS-CoV-2). The first case was reported in December 2019, in the city of Wuhan, in Hubei Province, China. Since then, COVID-19 has spread like a tsunami around the world and is now present in 213 countries and independent territories. According to the WHO, viral infections initiated by various coronaviruses continue to develop and pose a serious public health problem [13,14,15]. Distinctive features of the virus include its highly contagious nature and relatively long (1–14 days) development period. During this time, a person can become infected by the virus and show no symptoms. Therefore, people who have the disease can act as silent carriers of the virus without realizing it and contribute to a high number of basic reproductions of the COVID-19 virus. To date, there is no specific vaccine or treatment for infection, and management protocols focus on containing disease development. Most COVID-19 cases have exhibited clinical features such as fever, cough and fatigue. Some patients had symptoms such as headache, sore throat and shortness of breath, while symptoms such as runny nose, diarrhea, aches and pains were very rare, as shown in Table 2. While most COVID-19 patients develop mild to moderate disease, a few patients have been diagnosed with a severe (13.8%) and critical (6.1%) health condition [16]. According to the United States Centers for Disease Control and Prevention (CDC), people at the greatest risk of disease from COVID-19 are older adults (those over 60 years of age) and those with pre-existing conditions such as high blood pressure, asthma, diabetes, cardiovascular disease and those taking immunosuppressing therapy [17].

COVID-19 is categorized by a specific dysfunction in respiratory physiological processes involving the other parts of the lower respiratory tract and diaphragm, thereby affecting respiratory patterns during inhalation and exhalation from the lungs [18]. In speech initiation, at the time of exhalation, air from the lungs travels from other basic vocal subsystems, namely the larynx and trachea and the vocal canal into the oral, pharyngeal and nasal cavities. The way we breathe during speech, containing the speed and length of exhalation (depending on the number of words in a sentence or phrase), and its intensity and variability, greatly affect the quality of our voice. In addition, the respiratory system is primarily highly coordinated with these laryngeal-based subsystems [19,20]. Similarly, laryngeal activity is well linked to articulation in the oral and nasal cavities [21]. Although their effects and coordination of speech subsystems are perceptibly apparent with an inflammatory condition, these changes may be mild in the asymptomatic stages of a disease at baseline or recovery. Speech subsystems and coordination are assumed to be affected by COVID-19. In addition to the respiratory involvement by COVID-19, the current pandemic shows evidence that neurological involvement may occur with COVID-19. Headache and dizziness remain the most common symptoms; however, symptoms due to loss of muscle control and loss of proprioception have recently been reported due to transient neuromuscular disorder [22,23], as well as loss of smell and taste [24,25]. Given the physiological disorder to respiratory functions and evidence of this increased neurological issue due to COVID-19, biomarkers of COVID-19 derived from vocal subsystem coordination measurements are the most significant in the asymptomatic stage [26].

Although there are several studies in the direction of COVID-19′s pathophysiological properties, its propagation mechanism remains somewhat indefinable. While the initial COVID-19 cases were associated with the direct exposure of individuals to infected animals, the rapid outbreak of the disease has shifted the focus of the research to human-to-human via direct or other surface transmission. An analysis of around 75,465 cases of COVID-19 in China has revealed that the COVID-19 virus is primarily transmitted between people from the spread of respiratory droplets through coughing and sneezing [27]. These respiratory droplets have the potential to travel a distance of up to 1.8 m (6 feet). Therefore, any person in close contact with an infected person is at risk of being exposed to the respiratory droplets, and by extension, the virus. Although symptomatic people have been identified to be the primary source of SARS-CoV-2 transmission, there is also a possibility of transmission via asymptomatic people. Direct and indirect contact with infected surfaces have been identified as other potential causes of COVID-19 transmission. Evidence suggests that the virus can survive on plastic and steel surfaces. Researchers have revealed that COVID-19 is spread by contact. Therefore, it is recommended to minimize human-to-human contact for the safety of human society.

### 2.2. Human Activity Monitoring

Human activity monitoring plays an important role in human health. In the COVID-19 pandemic situation, it is essential to monitor human activities in terms of non-contact to stop the spread of the virus. Various non-contact human activity-sensing technologies, methods and performances achieved by existing platforms were investigated for the development of the COVID-19 platform. Device-free detection is a valuable technology for the detection of moving bodies in the operational region without the wearing of any device. The device-free passive detection of moving humans with dynamic speed (PADS) scheme extracts CSI with both types of information (amplitude and phase) and exploits space diversity across multi-antennas in multiple input multiple output (MIMO) systems. The prototype PADS uses commercial Wi-Fi devices to extract shape sensitive metrics for accuracy and robust target detection [28]. An active device-free system uses SDR to exploit activity recognition of a person standing, walking, crawling or lying and/or an empty environment [29]. Since wireless signals are good reflectors of human bodies, activities can be recognized by monitoring received Wi-Fi signals characteristics; CARM proposes a human activity recognition and monitoring system by extracting CSI and was implemented on commercial Wi-Fi devices [30]. A through the wall (TTW) presence detection system for both stationary and moving persons uses Wi-Fi signals with a single Wi-Fi access point (AP). This system considers an empty environment with one stationary human or a human moving in the room; the channel frequency response (CFR) changes over time carry significant information for monitoring activities [31]. Device free solutions based on radio signals (Wi-Fi) available in the home, particular 802.11 standard, have been considered. Fine-grained analysis based on available CSI have been proposed to detect human activities [32]. Human body motions were detected in a quasi-real-time environment using non-contact devices. Patterns of CSI present unique changes caused by body motions to identify particular human activities.

SDR technology has been exploited to extract a dataset that contains radio wave signals patterns [33]. Human activity recognition (HAR) using ultra-wide band (UWB) technology is very effective to investigate the feasibility of device-free activity recognition [34]. Hand gesture recognition is one of the issues in human–computer interactions. Non-contact Wi-Fi-based gesture recognition systems (Wi-GeRs) detect hand motions by capturing the changes in the CSI using Wi-Fi signals. A public Wi-Fi router is used for the detection of hand motions [35]. Non-contact sensing has attained a lot of attraction due to the availability of Wi-Fi signals in homes, offices, shopping malls, airports, etc. The commercial Wi-Fi infrastructure proposed a training-free human vitality sensing platform named Wi-Vit. This platform can capture real-time human motion speed information without the offline training or calibration that involves human effort. The feasibility study of the platform revealed that it can monitor long-term activities of daily living in practice for various applications [36]. Eating is an essential activity in human daily life. In this regard, a device-free system for the monitoring of eating uses Wi-Fi built-in devices (e.g., laptop or smartphone). This system automatically monitors human eating activities by extracting the fine-grained CSI from Wi-Fi signals of eating motions and by detecting swallowing and chewing. It can differentiate non-eating from eating activities and further classifies eating motions with different utensils. Eating monitoring is essential to understand eating behaviors, and it is useful for estimating a balanced diet [37]. The Wi-See system uses Wi-Fi signals for gesture recognition, since wireless signals can traverse TTW and do not require line-of-sight (LOS) from source to destination. The system uses wireless resources to enable entire-home gesture recognition. Wi-See was evaluated in a two-bedroom apartment and an office environment using SDR technology [38]. Wi-Hear uses Wi-Fi signals to “hear” human speech without installing any devices. This system introduces mouth motion profile (MMP) to solve micro movement detection problems that leverage wavelet packet transformation and partial multipath effects. It can “hear” human speech within the radio range, and it can simultaneously “hear” multiple human speech by exploiting MIMO technology. It was implemented on both commercial Wi-Fi infrastructure and the SDR platform [39].

An ambient radar sensor was proposed to recognize human activities in indoor environments. A radar uses 7.8 GHz frequency to capture the fine dynamics of human activities while emitting 16 pulse signals every second. This approach also includes a method to separate a collection of numerous activities into individuals [40]—the concept of domain gap (DG)—and further contains a domain independent (DI) feature, which is a promising solution to eliminate DG and achieve gesture recognition accuracy [41]. The Bumble-Bee radar captures micro-Doppler signatures for indoor human activity recognition. It can discriminate between human activities even under variable conditions [42]. Occupant activity recognition (OAR) is very important for building management systems (BMS) to give comfortable environments for occupants. The Wi-OAR system uses Wi-Fi signals to provide user-centric services and are energy-efficient in smart offices. This system presents a fast and robust target component separation (FRTCS) algorithm for measuring both high accuracy and time efficiency. A pair of commercial Wi-Fi devices was used for developing a prototyped Wi-OAR system in diverse office environments [43]. A public dataset by ten volunteers with sixteen different activities in indoor environments used Wi-Fi signals to develop the Wi-AR system. The aim of the system is to reduce the cost of collected signal data for researchers in a convenient manner and improve the performance in different domains [44]. Wi-Motion uses the amplitude and phase information extracted from the CSI sequence to build the classifiers. This system can recognize six different human activities [45]. A device-free, non-wearable, privacy-preserving occupancy detection system uses Wi-Fi imaging for future smart buildings. This system was developed using an off-the-shelf commercial Wi-Fi router, omnidirectional antennas and a network interface card (NIC) for imminent body-centric communication [46]. A low cost, non-intrusive and minimal low-power radar-based sensing system that uses a novel approach for human activity recognition in the home was developed that investigates fifteen different challenging activities performed inside the kitchen [47].

HAR uses deep learning (DL) networks with enhanced CSI to develop a CSI feature enhancement scheme (CFES). It includes two modules of background correlation feature enhancement and reduction for preprocessing the data input to the DLN [48]. At present, we are entering into the era of the Internet of Things (IoT), where it will be convenient to find APs at any location. The presence of a human body between two APs uses Wi-Fi signal waveforms to extract CSI. Machine learning (ML) uses CSI data to recognize and predict human motion [49]. With the popularization and development of Wi-Fi technology, it has become a benefit of human daily life to use mobile devices for monitoring daily activities [50]. Sleep monitoring is a very important human activity because it plays a key role in human health. Sleep-Guardian, a radio frequency (RF)-based healthcare system, combines signal processing, edge computing and ML [51]. Extensive running is life-threatening if it is not monitored properly. Wi-Run, a non-invasive step estimation, is a complete model-based system that intelligently estimates steps using commercial Wi-Fi devices. Wi-Run consisted of two models. The first model is the single runner CSI-based step estimation, which measures the relationship between single runner running and CSI dynamics in the activity area. The second model is the multi-runner CSI-based step estimation that quantifies the relationship between each runner’s running and CSI dynamics in the activity area [52]. Investigating spatial diversity in Wi-Fi signal-based HAR identifies the dead zones and their important dominant factors. A Wi-Fi signal-based spatial diversity aware non-contact activity recognition system (Wi-SDAR) was introduced. It overshadows the dead zones with only one physical Wi-Fi sender and receiver, which is fully compatible with commercial off-the-shelf Wi-Fi devices [53]. HAR uses radar as a sensor having unique characteristics such as contactless sensing and privacy protection. DL methods for activity recognition use radar to exploit human motion information [54].

**Figure 1:**
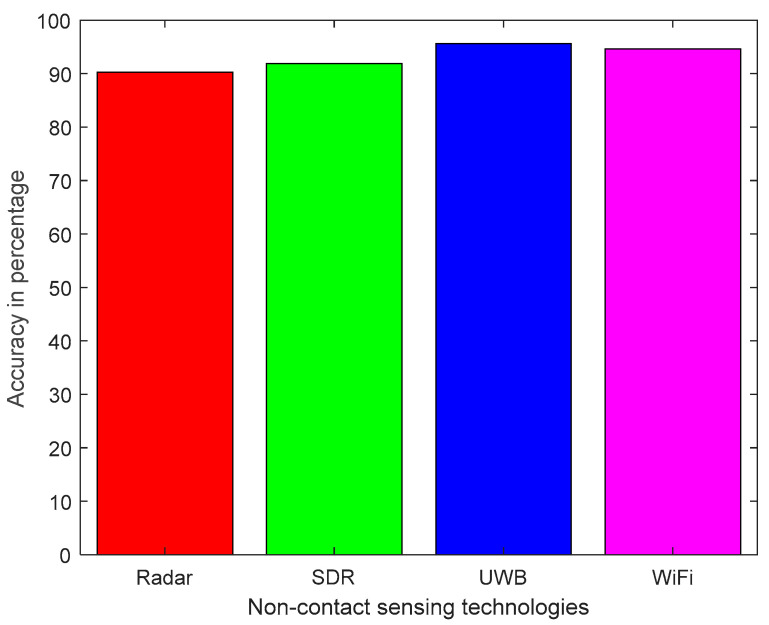
Average accuracy of non-contact sensing technologies for monitoring human activities.

Table 3 summarizes the non-contact sensing technologies, detection and monitoring, classification methods and accuracy achieved by existing research platform. Human activity classification and monitoring review reveals that non-contact sensing exploits WCSI to study human activities such as sitting, standing, walking, running, eating and sleeping. Wi-Fi sensing using commercial hardware is widely used because it is an inexpensive and easily available solution. Human activities have been monitored and classified in existing research by ML and DL algorithms having accuracies over 90% [28,30,31,32,33,34,35,36,37,38,39,41,42,43,44,45,46,47,48,49,50,51,52,53]. The average accuracy of promising non-contact technologies for monitoring human activities is shown in Figure 1.

### 2.3. Symptom Diagnosis and Health Monitoring

Regular health monitoring can detect potential health issues before they become a problem. In the COVID-19 pandemic, it is essential to diagnose early symptoms and monitor health conditions in a non-contact manner to stop the spread of the virus. Various non-contact sensing studies for monitoring health were investigated for the development of a COVID-19 platform. Wireless sensing was used to detect asthma attacks based on WCSI where Doppler effects were manifested [55]. The wireless sensing-based healthcare facility utilized 5 G C-band technology, which improved the efficiency of detecting the fall and body motions with a wide spectrum range and maximum capacity. The system works at 4.8 GHz frequency for capturing the WCSI of post-surgical falls and other important activities of humans. The low cost solution includes an RF signal generator, a NIC, and a desktop PC along with omni-directional antennas. This system is feasible and reliable for detecting post-surgical falls with high accuracy [56]. Huntington’s disease (HD) is a genetic disorder that cannot be cured easily. The quality of life of patients becomes more serious as the disease quickly progresses. It is essential to examine patients timely and effectively. A microwave sensing platform (MSP) was developed for continuous monitoring of HD patients in a non-contact manner. This platform also resolved patient inconvenience and privacy issues [57].

Parkinsonian gait is the most devastating symptom of Parkinson’s disease (PD), which has a more negative impact on quality of life than other PD symptoms. Wireless sensing technology is used for the detection of Parkinsonian gait using S-band for classification of normal walking and abnormal gait. Additionally, the early diagnosis of shaking palsy (SP) symptoms in a non-contact manner was also achieved [58]. Patients suffering from dementia show signs of wandering behavior due to memory loss or boredom. Dementia patients are exposed to serious injuries from falls if they are not continuously monitored. A wireless sensing platform was designed and evaluated the wandering behavior of patients suffering from dementia in an indoor environment [59]. Passive Wi-Fi sensing extracts the two-dimensional phase to monitor three health essentials, includes breathing rate, tremor and falls. The signal processing of the cross-ambiguity function (CAF) and various features are extracted from the signal [60]. Wireless sensing uses the C-band (4.8 GHz) in the indoor environment to monitor body movements of women, especially pregnant women, for early detection of seizure in pre-eclamptic women, so patients can be managed promptly and the mode of delivery can be decided early. The body movement shows unique features extracted from WCSI and can easily be differentiated by using ML classifiers [61]. A bathroom has a comparatively higher probability of severe accidents than other places or rooms due to a slippery floor. A commercial Wi-Fi device-based danger-pose detection system was used while preserving privacy [62]. A non-contact sensing method uses passive Doppler radar to capture human body movements to recognize respiration and other physical activities used for monitoring health. The system uses existing available wireless signals as a source to detect human activity. A two-stage signal processing framework was outlined to support the multi-purpose monitoring functionality. The first stage obtained the primary Doppler information by introducing high speed passive radar signal processing. The second stage functionality was signal processing of micro Doppler extraction data for breathing detection and classification [63].

Parasomnia is a sleep disorder that causes involuntary, random and unwanted movements of a dreaming patient. Unfortunately, these dreams may cause violent activities, which can result in more chances of injury, including that of a bed partner. Continuous monitoring of patients can prevent difficult situations. The system for continuous monitoring of patients exploits fine-grained magnitude and phase information of the WCSI. The variations in the WCSI, as a result of patient body movements, were monitored to identify the behavior [64]. Cerebellar ataxia (CA) is a neurological disease having symptoms of weak coordination movements and balance disorders. A non-contact sensing system was developed for detecting CA based on rapid alternating movements and heel–knee–shin diagnosis tests. This system has the potential to monitor CA in a flexible and patient-friendly environment [65]. A non-contact sensing method uses RF signals to detect paraparesis. It is a promising solution that can reduce the load and improve doctor work efficiency. A system used the 1D-convolution neural network (CNN) model for automatic extraction of valid features and classifications. The system performed efficient and accurate patient screening of suspected paraparesis [66]. Parkinson’s disease is a progressive neurologic disorder that primarily affects the movements and limits the motor ability of the patient. Freezing of gait (FOG) is a motor symptom of Parkinson’s disease in ageing people, and its timely treatment can reduce the probability of any secondary disorders. The magnitude and phase information of the radio signals is used to detect the motor and non-motor symptoms. The method is very useful with minimum deployment of resources for real-time patient monitoring systems [67].

Cerebellar dysfunction (CD) is one of several neurological disorders that disturbs the movement of the body. A user-friendly system was used to evaluate body movements in CD patients using S-band sensing technique. This system quantified the tremors in hand and gait abnormality using wireless devices such as a NIC, omnidirectional antennas and a router operating at 2.4 GHz to extract the CSI data [68]. Wireless sensor networks (WSNs) use directional antennas extensively for various applications. The four-beam patch antenna was used as a sensor node to evaluate the pill-rolling effect in Parkinson’s disease. The four-beam patch is highly directive, small in size and can mitigate the multipath fading present in an indoor environment for effective measurements. The pill-rolling affect indicates the tremors in the hands, predominantly in the fore-finger and the thumb. The developed system was a low-cost framework that evaluates the movement disorder using the S-band sensing platform leveraging wireless devices working at 2.4 GHz. The system efficiently classifies tremor and non-tremor feelings in the fingers [69]. A particular body movement of multiple sclerosis patients is monitored by a C-band sensing system working at 4.8 GHz, and especially the tremors and breathing patterns by a 5 G potential band. This system can identify the particular condition of a patient efficiently [70]. The wireless signal technology successfully detects human motions and related diseases in a non-contact manner [71]. Heart rate and breathing patterns of a person are major indicators of a physical condition. A system was developed for measuring the changes in the heart rate and breathing pattern of a person using commercial Wi-Fi devices. This is an inexpensive system and very useful for monitoring daily life health [72].

Human vital signs of heart rate and breathing along with body posture during sleep is very important to monitor and evaluate general physical health. A system was developed by using off-the-shelf Wi-Fi devices to track the vital signs of both heart rate and breathing rate during sleep without dedicated devices. An existing Wi-Fi network was re-used by the system to exploit the fine-grained CSI to capture every movement caused by heart beats and breathing. This system has the ability to monitor continuously and can be easily deployed everywhere with very cheap solutions [73]. FOG is a periodic absence of forward movement in PD patients, and it is one of the disabilities. A Wi-Freeze is a non-invasive Wi-Fi-based sensing system used for detection and classification of FOG [74]. The monitoring of various physical activities exploits wireless sensing devices, such as sensors used in medical cyber-physical systems (CPS). Patients undergoing epileptic seizures show signs of involuntary body movements. The system exploits S-band operating frequencies used for data extraction and classification of a clinical condition of epileptic seizures [75]. Wi-Fall is a system used for fall detection of independently-living people, especially the elderly. It can detect the fall of the human without any extra hardware setup or any wearable device. The system was implemented using commercial 802.11 n NIC. It can achieve high fall detection accuracy for a single person [76]. A real time (RT)-fall, contactless, inexpensive and accurate fall detection system used commercial Wi-Fi devices. It allowed users to perform routine activities continuously and naturally without attaching any devices on the body [77]. Res-Beat is a commercial Wi-Fi device-based system used for non-contact real-time respiration rate monitoring. The system analyzes bimodal CSI data for breathing signal anomalies to detect peak and estimate respiration rates [78].

The DL-based CNN model classifies ankle movements after surgery using the SDR platform. WCSI image data accurately detected movement of the ankle of patients who suffered fracture ankle surgery [79]. A non-contact sensing testbed was designed using universal software radio peripheral (USRP) devices for the classification of post-surgery activities. The testbed efficiently classified the weight lifting activity of spinal cord patients by exploiting WCSI [80]. Sometimes involuntary scratching may increase the spread of skin diseases such as atopic dermatitis. The frequency of scratching indicates the degree of itching and is helpful in analyzing clinical diagnosis. A system has the potential to monitor the scratching signal of a sleeping human body using a Wi-Fi router and a leaky coaxial cable (LCC) [81]. Hypopnea syndrome is a chronic respiratory disease that is described by repetitive occurrences of breathing disturbances during sleep. A contactless system provides an alternative to conventional medical testing for detecting incognito hypopnea syndrome using S-band wireless sensing. This system has the potential for monitoring accurate hypopnea syndrome in a user-friendly and flexible environment [82]. Respiratory rhythm is the indication of respiratory diseases. The ignored respiratory issues can be dangerous and may cause damage to other body tissues and organs. A non-contact respiratory rhythm detection used an MSP to capture the minute variations caused by breathing. This solution is affordable and its performance is high [83]. LCC has been used extensively in wireless communication to cover blind and semi-blind regions. A system used LCC to identify patient postures in bed in order to prevent or reduce bedsores. The indoor installation and periodic CSI data collection using 802.11 n Intel WLAN NICs helped to monitor postures [84]. A system monitored abnormal breathing patterns caused by sudden infant death syndrome (SIDS) and sleep apnea patients. This system used S-band wireless sensing to extract CSI data for the periodic and non-periodic signals that identify the normal and abnormal respiratory conditions [85]. Traditional, non-contact breathing detection systems required specialized hardware support that is not affordable in normal environments. Non-contact breathing detection systems based on C-band wireless sensing can easily be deployed in any environment. It is based on a multi-input, multi-output orthogonal frequency division multiplexing (MIMO-OFDM) system using 802.11 n protocol [86]. The focus of the 5 G autonomous network used wireless sensing for health care monitoring. The monitoring of respiratory symptoms for COPD (chronic obstructive pulmonary disease) used C-band wireless sensing to detect the respiratory conditions, including coughing and normal and abnormal breathing of a COPD patient by utilizing NIC and the CSI tool for the extraction of CSI with an omni-directional antenna operating at 4.8 GHz frequency. The 5 G sensing technology enhanced the health care system for detection of various diseases effectively [87].

**Figure 2:**
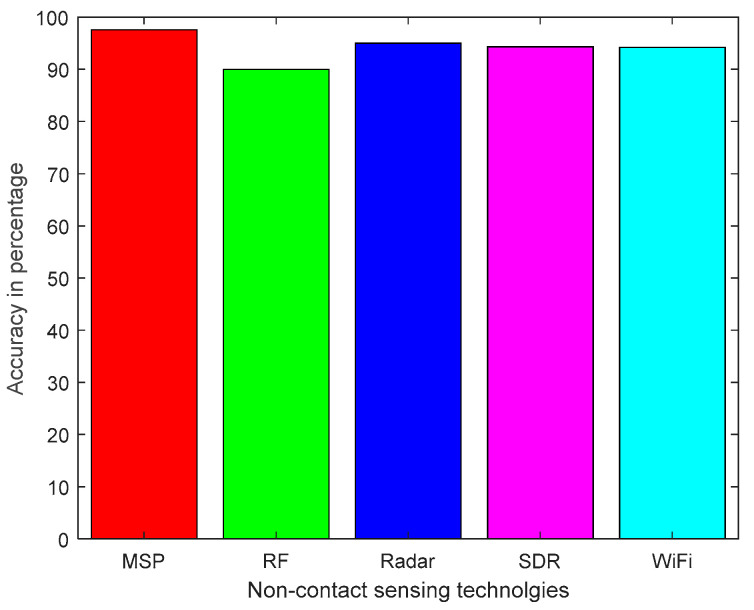
Average accuracy of non-contact sensing technologies for monitoring health.

Table 4 summarizes the non-contact sensing technologies, diagnosis of symptoms and monitoring health, classification method and accuracy achieved by the existing research platform. It was revealed from the literature review that the non-contact sensing approach has the potential for the early diagnosis of various symptoms to monitor health, such as breathing, heart rate, fall and sleep disorder. Most systems in the existing literature exploit Wi-Fi technology using CSI to detect and classify the health problems. The SVM algorithm is widely applied because it is applicable to both linear and non-linear data. The classification accuracy achieved by ML and DL algorithms is over 90% [55,56,57,58,59,60,61,62,64,65,66,67,68,69,70,71,72,73,74,75,76,77,78,79,80]. The average accuracy achieved by various non-contact sensing technologies to monitor health issues is shown in Figure 2.

## 3. Proposed Platform

**Figure 3:**
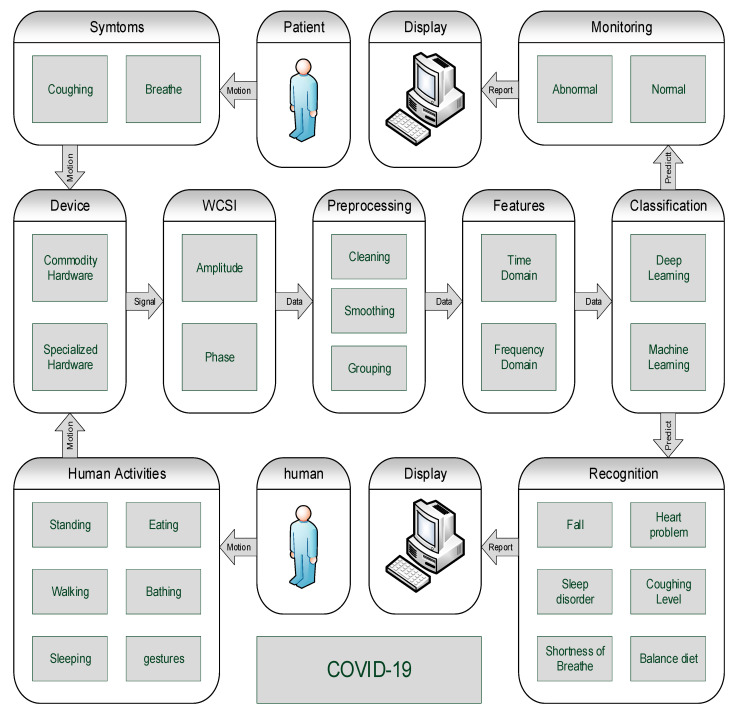
Non-contact sensing platform for diagnosis and monitoring.

A non-contact wireless platform is proposed on the basis of the existing literature. The five major functional blocks for the development of platforms are data collection devices, WCSI-based data extraction, data preprocessing, features extraction and classification, as shown in Figure 3.

### 3.1. Data Collection

The data can be collected by either specialize or commercial hardware devices. The coughing and breathing data are collected for early diagnosis of COVID-19 symptoms. The data of sitting, standing, walking, sleeping, eating and posture are collected for the monitoring and detection of fall, heart rate, sleep disorder and diet to protect human lives in COVID-19 pandemic.

### 3.2. Data Extraction

The OFDM signal is used for fine grained WCSI extraction at the receiver. The WCSI frequency response of each activity is monitored continuously, having the information of the number of sub-carriers, the number of samples and the time taken to complete the activity. The time and samples can be expressed as the number of samples received in a unit time. This sampling time can be chosen on the basis of the device sample rate. The total frequency response of WCSI is express in Equation (1):

(1) $$H{\left(j\omega \right)}_{total}=\left[\begin{array}{cccc} H_{11} & H_{12} & \ldots & H_{1s}\\ H_{21} & H_{22} & \ldots & H_{2s}\\ \vdots & \vdots & \ldots & \vdots \\ H_{k1} & H_{k2} & \ldots & H_{ks} \end{array}\right]$$

where *k* represents the maximum number of sub-carriers, and *s* represents the total number of samples. The WCSI frequency response of single OFDM frame can be expressed as in Equation (2):

(2) $$H{\left(j\omega \right)}_{sub-carrier}=\left[H\left(j\omega_{1}\right),H\left(j\omega_{2}\right),\ldots H\left(j\omega_{k}\right)\right]$$

The WCSI frequency response of each sub-carrier contain amplitude and phase information, it can be expressed as in Equations (2) and (3), respectively:

(3) $$\left|H\left(j\omega_{k}\right)\right|=\;\sqrt{H{\left(j\omega_{k}\right)}_{real}{}^{2}+H{\left(j\omega_{k}\right)}_{img}{}^{2}}$$

(4) $$∠H\left(j\omega_{k}\right)=\;-tan^{-1}\left(\frac{H{\left(j\omega_{k}\right)}_{img}}{H{\left(j\omega_{k}\right)}_{real}}\right)$$

$\left|H\left(j\omega_{k}\right)\right|$ is the amplitude of the kth subcarrier, and $∠H\left(j\omega_{k}\right)$ is the phase of the kth sub-carrier. The amplitude and phase information of WCSI is useful for identifying the human body motion to recognize human activity and health condition.

### 3.3. Preprocessing

Data preprocessing requires data cleaning, smoothing and grouping to ensure meaningful, accurate and efficient analysis. Data cleaning is a process to remove and replace missing or bad data. It detects abrupt changes and local extrema, which is useful to find significant data trends. The smoothing process remove noise using filtering and other signal processing techniques. The grouping process is used to identify correlations among the data values.

### 3.4. Features

Feature extraction is a transformation of information, which changes measured data into meaningful information. In addition, it is a dimension reduction process to reduce the computation complexity and time. Presently, statistical characteristics approaches have been used for feature extraction. In the literature, various features are extracted on the basis of data properties to improve the classification performance. According to WCSI data properties, statistical features are divided into two categories, such as the time-domain and frequency-domain [11,88,89,90]. The most important features used for WCSI data are listed in Table 5.

### 3.5. Classification

Most human activity recognition approaches exploit the ML and DL algorithms to classify the motion type and its corresponding human activity to test the health status. The efficiency of the classifier depends on the type of dataset. ML can be used to develop activity detection models that make health predictions based on WCSI data in the presence of uncertainty. Adaptive algorithms classify normal and abnormal health patterns in the WCSI data. When a learning computer is exposed to more experimental data, the computer improves its identification performance. The entire set of WCSI data is considered as a heterogeneous matrix. The WCSI response data set is a column vector where each row is labeled with the corresponding activity in the WCSI row data. ML model accuracy is used as a diagnostic measure to reflect the validated model results [80]. In DL, CNN learns useful information from images. In the existing literature, it is used for monitoring purposes in many research studies. Currently, DL is efficiently applied in the biomedical area. AlexNet and ZFNet are the most popular CNN architectures and can be used in a parallel manner to classify WCSI numeric data that is converted to images [79].

## 4. Experimental Setup

On the basis of the literature review, an experimental setup is proposed for the development of a COVID-19 platform. In the following, two hardware setups are proposed to conduct experiments in a bedroom along with a bathroom to capture RT environments.

### 4.1. Commercial Hardware Platform

**Figure 4:**
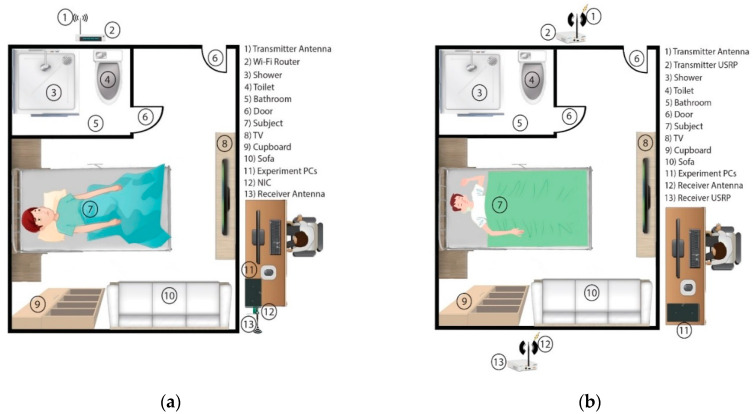
Proposed experimental setup: (**a**) commercial hardware platform; (**b**) specialize hardware platform.

This hardware platform consists of a Wi-Fi router, NIC, desktop PC or laptop and omni-directional antennas, as shown in Figure 4a. It utilizes a CSI tool inbuilt on the Intel Wi-Fi wireless link (IWL) 802.11 n MIMO radio, which uses an open source Linux wireless driver and custom modified firmware. It includes all the software to read and parse the channel measurements and scripts needed to run experiments. The IWL provides 802.11 n standard WCSI in a data format that reports the channel matrices of 30 sub-carrier groups, which is about one group for every 2 sub-carriers at 20 MHz or one group for every 4 sub-carriers at 40 MHz frequency. Each channel matrix entry is a complex number, with signed 8-bit resolution, each for the real and imaginary parts. It specifies the gain and phase of the signal path between a single transmit–receive antenna pair. The hardware setup is inexpensive, easily accessible and commercially available [91,92].

### 4.2. Specialized Hardware Platform

This hardware platform consists of two USRP devices, one for transmission and the other for reception, along with omni-directional antennas and a desktop PC or laptop, as shown in Figure 4b. Currently, different SDR platforms are used for experimental research; among them, the USRP manufactured by Ettus research is mostly used, which has become the standard choice for wireless research [93,94]. The architecture incorporates the Xilinx Spartan-6 FPGA along with the agile analog radio frequency integrated circuits (RFIC)’s direct adaptation transceiver. The RFIC determines the number of independent transceivers. It integrates independent coherent transceivers that allow implementation of *n* × *n* MIMO systems. This device can cover a wide range of frequency coverage with an adjustable bandwidth and can run in frequency division duplex (FDD) or time division duplex (TDD) mode; it allows the FDD to operate in full-duplex mode, while the TDD operates in half-duplex mode [95]. This hardware setup is flexible and portable.

Various experiments can be carried out on commercial and specialized hardware platforms to develop fully functional non-contact sensing COVID-19 platform. Experiments are to be divided into two main categories:

1. Symptoms data collection

Initially, coughing and shortness of breath data are collected from patients for developing a ML/DL model to classify suspected patients of COVID-19. From the literature review [40,45,48,57,63,70], both coughing and breathing can be monitored in a non-contact manner.

2. Activities data collection

It is very essential to recognize human activities for stability during the isolation and quarantine period. Standing, walking, sleeping, eating, bathing and postures data are collected for developing a ML/DL model to recognize the fall, sleep disorder and diet of the patient. From the literature review, heart problems, fall, sleep disorder and eating habits can be recognized by non-contact wireless sensing platforms [14,15,17,20,22,23,25,26,27,28,30,31,33,34,36,38,40,45,47,48,49,57,61,62,63].

## 5. Outcomes

The following are the outcomes from the development of a non-contact sensing platform for containing of COVID-19.

1. Wireless signals can pass through the wall and do not require LOS. This feature of non-contact sensing eliminates the need for face-to-face contact and provides improved management to contain COVID-19.
2. In case COVID-19 symptoms develop, the data sent by means of cloud computing platforms can enable healthcare authorities to respond quickly.
3. It will reduce the physical contact time with a COVID-19 patient as much as possible.
4. It will not only monitor COVID-19 symptoms but also continuous health monitoring during quarantine and isolation periods in a non-contact manner.
5. Transferring care to home, or treating high-risk elders and children in their own homes.
6. This will improve privacy of individuals during quarantine or isolation periods.
7. It will also help in early recognition of patients who need aggressive management or hospitalization to prevent them from serious or irreversible sequelae of the disease.
8. Reduce life risk of doctors, paramedical staff and caretakers during quarantine and isolation periods.
9. Innovative tools to construct useful contactless sensing platforms for health care applications.
10. These platforms can be deployed by re-using the existing infrastructure of wireless communication networks.
11. Improved access to care, increased quality of care and reduced care costs.
12. It can be deployed in any emergency condition at any place to counter health challenges.

## 6. Challenges

Although the literature review has demonstrated the potential capabilities for developing a non-contact sensing platform for the monitoring of COVID-19 to contain the virus and protect humanity, there are still existing challenges and research problems that need further investigation and exploration.

*A.*  *Environmental effect*

The real time environment is challenging for developing a classification model using WCSI. The environment varies from place to place due to furniture movement, closing and opening of windows and doors, electronic devices, etc., which leads to changes of the behavior of the wireless channel. It is necessary to develop a model which can adopt to new environment.

*B.*  *Experimental subject*

One of the biggest challenges in data collection is the subject used in the medical related experiments. It is very difficult to use real patients in all the experiments. Diseases and health status vary greatly from patient to patient and during different time periods. With COVID-19, early symptoms also vary from patient to patient. On the other hand, experimenting with real patients is not comfortable for them and also requires time to perform extensive experiments. Researchers mainly used healthy subjects for performing experiments, which may not address the actual problem.

*C.*  *Orientation and location*

The user’s orientation and location also have critical effects on the performance of WCSI-based sensing systems. The differences in users’ orientation and location may cause different variations in WCSI measurements. Existing research mainly used the same orientation and location during experiments. However, a few research studies considered different orientations and locations to overcome such limitations.

*D.*  *Multi-subject sensing*

Most of the research on WCSI-based sensing platforms considered a single subject for investigation. It is challenging to differentiate the movements of multiple subjects using WCSI measurements. Considering the COVID-19 isolation or quarantine period guidelines, a single subject is enough for developing the platform. However, early diagnosis of COVID-19 symptoms requires multi-subject sensing, because before diagnosing COVD-19 as positive, people are not quarantined from their families and are living in a multi-subject environment. This stage is difficult to monitor and is the main source of the viral spread.

*E.*  *Privacy and security*

With the rapid expansion of Wi-Fi sensing technology, it also raises privacy and security issues. Existing research has demonstrated that Wi-Fi signals can interfere with other users. Enemies may spy the activities and position using existing human activity sensing systems. It is necessary to pay more attention to the improved privacy and security concerns.

## 7. Future Recommendations

The following are the future recommendations to improve the non-contact sensing platform for the monitoring of human activities and health conditions to contain COVID-19:

1. It is recommended to perform extensive experiments with different environments and experimental setups to develop an RT model.
2. It is recommended to collect experimental data by using multi-subjects with extensive experimentation to develop a model.
3. An efficient and possible solution must develop a rigorous theoretical model independent of the user’s location and orientation; the correct mapping of the relationship between WCSI measurements and the human body motions identify the health conditions. It is recommended to conduct experiments with different orientations and locations for the collection of data for developing models.
4. It is recommended to extract more prominent features to differentiate human activities and health conditions. Frequency domain features are useful for classifying multi-subjects.
5. It is recommended to use SDR-based WCSI sensing to counter the privacy and security using a self-generated signal approach that can switch to different frequency bands.

## 8. Conclusions

Various measurements and research studies are initiated to contain COVID-19 throughout the world. Limiting human-to-human contact is the best solution to reduce the spread of COVID-19. This research presents a comprehensive review on existing non-contact sensing of human activities and health monitoring that could be used for the development of a COVID-19 pandemic platform. The Wi-Fi and SDR technology has the potential to contain COVID-19 in a non-contact manner. This study proposes a non-contact WCSI-based sensing platform for monitoring COVID-19 to contain the deadly pandemic situation.

The proposed platform has the potential to diagnose the early symptoms like coughing and shortness of breath. The development of the platform is very useful in the quarantine and isolation period because it will monitor fall, sleep disorder, shortness of breathing, coughing level, heartbeat and diet of suspected or confirmed COVID-19 cases. Although the proposed platform is a promising solution, there still exist limitations to achieve optimal performance. This study highlights the challenges, and it is expected that proposed solutions will contribute to contain COVID-19.

## Figures and Tables

**Table 1:** List of abbreviations used.

Abbreviation	Description	Abbreviation	Description
AP	Access point	MIMO	Multiple input multiple output
BMS	Building management systems	ML	Machine learning
CA	Cerebellar ataxia	MMP	Mouth motion profile
CAF	Cross-ambiguity function	MSP	Microwave sensing platform
CARM	Channel state information-based human activity recognition and monitoring	NIC	Network interface card
CD	Cerebellar dysfunction	OAR	Occupant activity recognition
CDC	Centers for Disease Control and Prevention	OFDM	Orthogonal frequency Division multiplexing
CFES	CSI feature enhancement scheme	PADS	Passive detection of moving humans with dynamic speed
CFR	Channel frequency response	REM	Rapid eye movement
CNN	Convolutional neural network	RF	Radio frequency
COPD	Chronic obstructive pulmonary disease	RFA	Random forest algorithm
COVID	Corona virus disease	RFIC	Radio frequency integrated circuits
		RNN	Recurrent neural network
CSI	Channel state information	RT	Real time
DG	Domain gap	SARS-CoV-2	Severe acute respiratory syndrome coronavirus-2
DI	Domain independent	SDAR	Spatial diversity aware non-contact activity recognition
DL	Deep learning	SDR	Software defined radios
DTW	Dynamic time warping	SIDS	Sudden infant death syndrome
EWMA	Exponentially weighted moving average	SP	Shaking palsy
FDD	Frequency division duplex	SSF	Stable signal fusion
FDTW	Fast dynamic time warping	SVM	Support vector machine
FOG	Freezing of gait	TDD	Time division duplex
FPGA	Field programmable gate array	TTW	Through the walls
FRTCS	Fast and robust target Component separation	USRP	Universal software radio peripheral
HAR	Human activity recognition	UWB	Ultra-wide band
HD	Huntington’s disease	WCSI	Wireless channel state information
HMM	Hidden Markov model	Wi-AR	Wireless activity recognition
IoT	Internet of things	Wi-Fi	Wireless fidelity
IWL	Intel Wi-Fi wireless link	Wi-GeR	Wi-Fi-based gesture recognition
KNN	K-nearest neighbors	Wi-Hear	Wireless Hear
LCC	Leaky coaxial cable	Wi-Motion	Wireless Motion
LOS	Line of sight	Wi-See	Wireless See
LSTM	Long short term memory	Wi-Vit	Wireless Vitality
MCP	Medical cyber–physical	WSN	Wireless sensor networks

**Table 2:** Summary of COVID-19 symptoms.

Symptoms		COVID-19
Fever	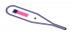	Most common
Cough	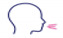	Most common
Sore throat	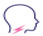	Less common
Shortness of breath	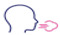	Less common
Fatigue	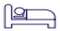	Most common
Aches and pains	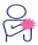	Rare
Headaches	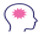	Most common
Runny or stuffy nose	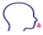	Rare
Diarrhea	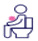	Rare

**Table 3:** Summary of existing contributions in human activity classification and monitoring.

Sr	Technology/Reference	Detection and Monitoring	Classification Method	Accuracy
1	Wi-Fi sensing [28]	Moving human	SVM	99%
2	SDR [29]	Standing, walking, crawling, lying and empty	KNN	85%
3	Wi-Fi sensing [30]	Walking, running sitting and falling, opening, empty, refrigerator, boxing, pushing one hand, brushing teeth	CSI-speed and CSI-activity model	96%
4	Wi-Fi sensing [31]	Human presence static and dynamic	Naïve Bayes	99%
5	Wi-Fi sensing [32]	Walk, Sit, Stand, Run	SVM and LSTM	95%
6	SDR [33]	Standing up or sitting down	RF	96.70%
7	UWB [34]	Standing, sitting, lying	RF	95.6%
8	Wi-Fi sensing [35]	Gestures	FDTW	97.28%
9	Wi-Fi sensing [36]	Human motion	HMM	94.2%
10	Wi-Fi sensing [37]	Eating	Soft decision-based learning	95%
11	SDR [38]	Gestures	Pattern-matching	94%
12	SDR and Wi-Fi sensing [39]	Hearing	ML	91%
13	Radar sensing [40]	Sit-to-stand, stand-to-sit, walking and jogging	K-mean	85%
14	Wi-Fi sensing [41]	Gestures	CNN	94.45%,
15	Radar sensing [42]	Walking, running, and crawling	KNN	93%
16	Wi-Fi sensing [43]	Whole-body movements and partial-body, seated activities	ML	94.82%
17	Wi-Fi sensing [44]	Upper, Lower and whole body	CNN	90%
18	Wi-Fi sensing [45]	Bend, hand clap, walk, phone call, sit down and squat	SVM	98.4%
19	Wi-Fi sensing [46]	Pick up, walking, jogging and sitting on chair	Deep auto-encoder	91.1%
20	Radar sensing [47]	Kitchen activities	CNN	92.8%
21	Wi-Fi sensing [48]	Standing and stand-up, sitting and sit down	Soft-max regression	97.5%
22	Wi-Fi sensing [49]	Walk, stand, empty and sit down	RNN	90%
23	Wi-Fi sensing [50]	Moving area, path walking	Path matching	90.83%
24	Wi-Fi sensing [51]	Sleep	K-NN	93.88%
25	Wi-Fi sensing [52]	Quantifying running	SSF	93.18%
26	Wi-Fi sensing [53]	Walking	STFT	96%

**Table 4:** Summary of existing contributions in diagnosis of symptoms and monitoring of health.

Sr	Technology/Reference	Detection and Monitoring	Classification Method	Accuracy
1	RF sensing [55]	Asthma attacks	SVM	90%
2	Wi-Fi sensing [56]	Post-surgical fall	SVM	90%
3	Wi-Fi sensing [57]	Huntington’s disease	SVM and RF	98%
4	Microwave spectrum sensing [58]	Parkinsonian gait	SVM	94%
5	Wi-Fi sensing [59]	Dementia	SVM	90%
6	SDR [60]	Breathing rate, tremor and falls	ML	98%
7	Wi-Fi sensing [61]	Eclamptic seizures	SVM	95%
8	Wi-Fi sensing [62]	Danger-pose	SVM	96.23%
9	SDR [63]	Breathing	SVM	85%
10	SDR [64]	REM sleep disorder	SVM	90%
11	Microwave sensing [65]	Cerebellar ataxia	SVM	99.8%
12	Wi-Fi sensing [66]	Paraparesis	1D-CNN	99.4%
13	Microwave sensing [67]	Neurological disorder	SVM	99%
14	Wi-Fi sensing [68]	Cerebellar dysfunction	SVM	91%
15	Wi-Fi sensing [69]	Parkinson’s disease	SVM	90%
16	Wi-Fi sensing [70]	Tumor	SVM	90%
17	Radar sensing [71]	Gait	SVM	95%
18	Wi-Fi sensing [72]	Breathing and heart rate patterns	DTW	94%
19	Wi-Fi sensing [73]	Vital sign during sleep	SVM and RF	93%
20	Wi-Fi sensing [74]	Parkinson’s disease	CNN	99.7%
21	RF sensing [75]	Epileptic seizures	SVM	90%
22	Wi-Fi sensing [76]	Fall	SVM and RF	94%
23	Wi-Fi sensing [77]	Fall	SVM	100%
24	Wi-Fi sensing [78]	Respiration rate	EWMA	93.04%
25	SDR [79]	Post-surgery ankle fractured	CNN	98.98%
26	SDR [80]	Post-surgery spinal cord	FKNN	99.6%

**Table 5:** Time and frequency domain statistical features.

Time Domain Features				Frequency Domain Features	
Statistics	Expression	Statistics	Expression	Statistics	Expression
Minimum	$Min\left(X_{i}\right)$	Skewness	$\frac{1}{N}\overset{N}{\underset{i=1}{\sum }}{\left(\frac{x_{i}-u_{x}}{\sigma }\right)}^{3}$	FFT	$FFT\left(d\right)=\overset{N}{\underset{n=-N}{\sum }}x\left(n\right)e^{-j\frac{2\pi }{N}nd}$
Maximum	$Max\left(X_{i}\right)$	Kurtosis	$\frac{1}{N}\overset{N}{\underset{i=1}{\sum }}{\left(\frac{x_{i}-u_{x}}{\sigma }\right)}^{4}$	Spectral probability	$p\left(d\right)=\frac{FFT{\left(d\right)}^{2}}{\sum_{i=-N}^{N}FFT{\left(i\right)}^{2}}$
Mean	$\frac{1}{N}\overset{N}{\underset{i=1}{\sum }}x_{i}$	Histogram	$Histogram\left(x_{i}\right)$	Signal energy	$E=\overset{N}{\underset{n=-N}{\sum }}{\left|p\left(d\right)\right|}^{2}$
Variance	$\overset{n}{\underset{i=1}{\sum }}{\left(x_{i}-u_{x}\right)}^{2}$	Interquartile range	$Q_{3}-Q_{1}$	Spectrum entropy	$H=\overset{N}{\underset{i=-N}{\sum }}p\left(d\right)\ln\left(p\left(d\right)\right)$
RMS	$\sqrt{\frac{1}{N}\overset{N}{\underset{i=1}{\sum }}x_{i}{}^{2}}$	Range	$x_{max}-x_{min}$	Frequency peak	Max(FFT(d))
